# Laparoscopic pyeloplasty for ureteropelvic junction obstruction of the lower moiety in a completely duplicated collecting system: a case report

**DOI:** 10.1186/1752-1947-2-333

**Published:** 2008-10-22

**Authors:** Konstantinos G Stravodimos, Ioannis Anastasiou, Ioannis Adamakis, Theodoros Kapetanakis, Georgios Koritsiadis, Constantinos Constantinides

**Affiliations:** 1 1st Urology Department, 'Laiko' General Hospital, University of Athens Medical School, Athens, Greece; 2 49, Thisseos st, P. Faliro, 175 62 Athens, Greece

## Abstract

**Introduction:**

There are only a few reports on laparoscopic pyeloplasty in kidney abnormalities and only one case for laparoscopic pyeloplasty in a duplicated system. Increasing experience in laparoscopic techniques allows proper treatment of such anomalies. However, its feasibility in difficult cases with altered kidney anatomy such as that of duplicated renal pelvis still needs to be addressed.

**Case presentation:**

We present a case of a 22-year-old white Caucasian female patient with ureteropelvic junction obstruction of the lower ureter of a completely duplicated system that was managed with laparoscopic pyeloplasty. Crossing vessels were identified and transposed. The procedure was carried out successfully and the patient's symptoms subsided. Follow-up studies demonstrated complete resolution of the obstruction.

**Conclusion:**

Since laparoscopic pyeloplasty is still an evolving procedure, its feasibility in complex cases of kidney anatomic abnormalities is herein further justified.

## Introduction

Despite the emergence of endoscopic techniques and the recent development of laparoscopic approaches, many patients with ureteropelvic junction (UPJ) obstruction are still managed through open pyeloplasty. It is probably because of technical difficulties and the need for intracorporeal knot tying that this procedure has not yet been adopted worldwide as the initial treatment of choice in UPJ obstruction. However, increasing experience has gradually identified laparoscopic pyeloplasty as the optimum procedure since it combines success rates similar to that of open surgery with the low morbidity of laparoscopic approaches [1]. Both open and laparoscopic pyeloplasty report success rates over 90%. The latter is superior to endoscopic techniques which cannot address the extrinsic causes of UPJ obstruction. If one takes into account the advantages of laparoscopic surgery, laparoscopic pyeloplasty could be expected to dominate in the forthcoming years as a novel standard approach in UPJ obstruction.

Since the efficacy of laparoscopic pyeloplasty for UPJ obstruction co-existing with a duplicated collecting system has been reported in one adult patient [2], a second case is herein reported.

## Case presentation

**Figure 1:**
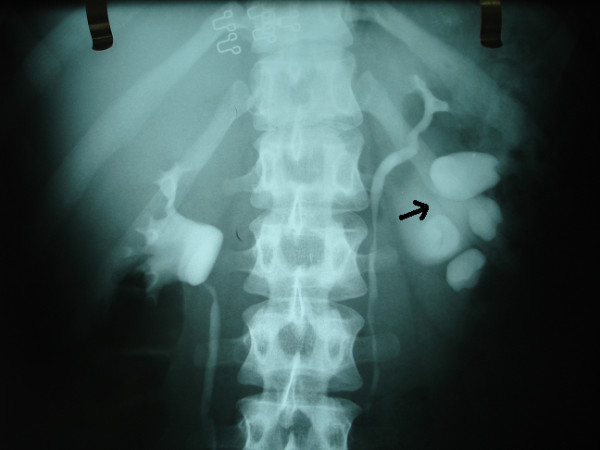
Pre-operative intravenous pyelography; ureteropelvic junction obstruction in the lower of the two collecting systems.

The patient, a 22-year-old white Caucasian woman, presented with a long history of recurrent left colicky flank pain. An ultrasound scan revealed a dilated left kidney. Intravenous pyelography studies indicated left renal pelvis duplication with concomitant ureter duplication. Furthermore, UPJ obstruction was apparent in the lower of the two systems (Figure 1). Despite the presence of outflow obstruction, the kidney's functional capacity was only moderately decreased implying that prompt obstruction relief would likely result in complete remission and a fully functional kidney. After extensive counseling and discussion of the various therapeutic options and likely outcomes with the patient, pyeloplasty was chosen. Previous experience with laparoscopic surgery, the patient's young age and the already reported success of the procedure in the upper pole of a duplicated collecting system, pointed toward a laparoscopic rather than an open approach.

### Surgical technique

After general anesthesia induction, the patient received a single dose of intravenous second-generation cephalosporin and a second one 8 hours postoperatively. In addition, subcutaneous heparin was also administered at the same time, and a urethral Foley catheter was placed.

The patient was then placed in a lateral decubitus position and was ready to be securely stabilized on the operating table. Using the Hasson technique, pneumoperitoneum was established by insertion of a 10 mm port, 2 cm laterally to the umbilicus. A three-port technique was utilized and two additional laparoscopic ports were inserted below the costal margin and at the ipsilateral lower quadrant along the midclavicular line. After Toldt line incision, the duplicated UPJ was identified along with the two ureters located medially to the lower pole of the kidney.

After cranial mobilization of the lower ureter, accessory crossing vessels were identified at the level of the UPJ. Crossing vessels ran anteriorly to both ureters which originated from the upper and lower moiety, respectively. During the procedure, the normal upper moiety ureter was identified and protected from inadvertent accidental injury. After mobilization of the renal pelvis, the UPJ was circumferentially transected, the ureter spatulated towards the lower pole of the kidney over 2 cm and the renal pelvis reduced appropriately. Before the initiation of the anastomosis, the ureter was transposed to the opposite (anterior) side of the crossing vessels. A classic Hynes-Anderson dismembered pyeloplasty was performed using two 4-0 absorbable running sutures for both anterior and posterior anastomosis. Intracorporeal knot tying was performed in a free-hand fashion. When the posterior part of the anastomosis was concluded, a guidewire was inserted through a trocar in the ureter reaching the bladder and antegrade stenting (using a double J stent) was performed. A 14F suction drain was finally placed through one of the port sites.

**Figure 2:**
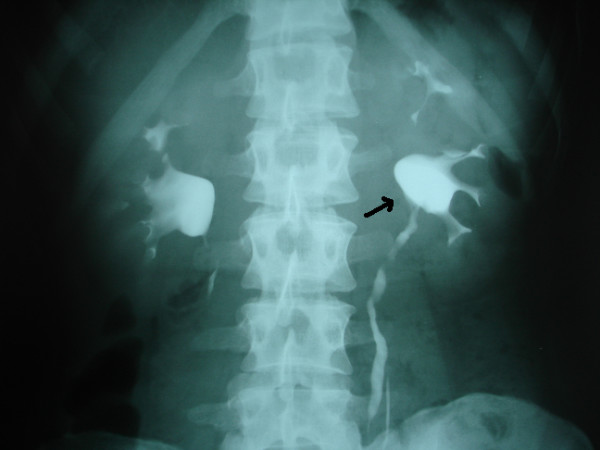
Postoperative intravenous pyelography; normal ureteropelvic junction.

Overall, operative time was 260 minutes and blood loss was minimal (approximately 30 ml). The drain was free of output on the third postoperative day, when it was removed and the patient discharged. Four weeks postoperatively, the ureteral stent was removed. Currently, 18 months postoperatively, the patient is doing well and is free of any symptoms. Intravenous pyelography studies at 6 months revealed a normal UPJ, free of signs of obstruction (Figure 2).

## Discussion

Even though open pyeloplasty remains the standard of treatment for UPJ obstruction management with success rates exceeding 90% [3,4], recent advances in laparoscopic surgery have led an increasing number of surgeons to adopt minimally invasive approaches. Laparoscopic pyeloplasty constitutes the ambitious counterpart of the open procedure with its first report dating back to 1993 [5] when Schuessler and co-workers reported on the feasibility of laparoscopic transperitoneal dismembered pyeloplasty in a series of five patients with UPJ obstruction. Since then, major advances in endoscopic techniques and surgical training have been made and larger series [4] have claimed success rates similar to that of the open approach, questioning its suitability as the standard of care. Major drawbacks in laparoscopic pyeloplasty utilization include its technical complexity and the duration of the operation. To this very day, the task of intracorporeal knot-tying renders laparoscopic pyeloplasty a challenging procedure, which is probably the reason why, within a decade, the largest series are up to 100 patients and are coming from high expertise centers [6].

Further to its widespread utilization in large series, laparoscopic pyeloplasty has been reported to be efficient in difficult cases such as persistent UPJ obstruction after failed open pyeloplasty and some kind of salvage endoscopic approach [7]. Bove and co-workers have also reported on the viability of the procedure in an 11-patient series with upper urinary tract abnormalities rendering them complex, one of whom was found to have a duplicated collecting system [8].

Despite the efficacy of laparoscopic pyeloplasty in UPJ obstruction in our patient, other techniques are also feasible. Kumar and colleagues [9] recently reported on the feasibility of laparoscopic pyeloureterostomy in a similar case with excellent results. This experience, along with other reports [10], indicate laparoscopic pyeloureterostomy as a viable alternative which should also be considered in cases of UPJ obstruction especially in incomplete duplicated collecting systems (also treating the yo-yo reflux presenting in these systems). Since our case involved a complete ureteral duplication without reflux and with normal upper moiety, a pyeloplasty was performed.

In addition to this series of reports we herein add a confirmation of laparoscopic pyeloplasty as a feasible option for management of adult UPJ obstruction in a completely duplicated collecting system. The case presented is novel in that it reports on the efficacy of laparoscopic pyeloplasty in the lower segment of a duplicated system in the presence of crossing vessels. In their report on UPJ obstruction management in a duplicated collecting system, Sahai and co-workers [2] did not confront crossing vessels pre- or intra-operatively. As with most authors, we preferred dismembered pyeloplasty (Hynes-Anderson) via a transperitoneal approach, the latter allowing easy identification and mobilization of intra-abdominal structures and adjacent viscera.

Further, compared to the report of Sahai and colleagues [2], our case is also different in that we confronted a completely normal upper moiety (versus a massively dilated upper and a less dilated lower moiety). In addition, Sahai et al. described an anastomosis near the confluence of the pelvic systems with an originating single ureter. In comparison, our case demonstrates an anastomosis in one of two completely independent pelvic systems.

After surgery, the patient's subjective symptoms as well as imaging studies have significantly improved arguing in favor of the procedure's success. Postoperative course and final cosmetic result were also excellent, an issue of importance for a young female patient.

## Conclusion

In conclusion, we argue for the feasibility of laparoscopic pyeloplasty for UPJ obstruction management in a duplicated collecting system, also in the setting of co-existing crossing vessels. This report further supports the trend of endoscopic surgery utilization even in cases complicated by modified renal anatomy.

## Abbreviations

UPJ: ureteropelvic junction.

## Competing interests

The authors declare that they have no competing interests.

## Authors' contributions

KGS was the main surgeon and made substantial contribution to conception and design, provided all operative details and photographic material in the present work while substantially contributing to manuscript preparation and revision. IA contributed in patient care, acquisition of data and has been involved in drafting the manuscript. IA, TK, GC contributed in patient care, data analysis, literature review and multiple revisions of the manuscript while CC cooperated in design, and critically revising of the final version of the manuscript. TK was especially involved with the initial draft as well as final draft corrections and correspondence. All authors have given final approval of the version to be published.

## Consent

Written informed consent was obtained from the patient for publication of this case report and any accompanying images. A copy of the written consent is available for review by the Editor-in-Chief of this journal.
